# Delayed Deep White Matter Ischemia After Resection of Gliomas by Awake Surgery

**DOI:** 10.1227/neuprac.0000000000000105

**Published:** 2024-08-28

**Authors:** Takahiro Tsuchiya, Masamichi Takahashi, Makoto Ohno, Shunsuke Yanagisawa, Sho Osawa, Shohei Fujita, Yoshitaka Narita

**Affiliations:** Department of Neurosurgery and Neuro-Oncology, National Cancer Center Hospital, Tokyo, Japan

**Keywords:** Awake craniotomy, Deep white matter, Glioma resection, Ischemia, Watershed lesion

## Abstract

**BACKGROUND AND OBJECTIVES:**

Deep white matter (DWM) is perfused by the medullary arteries from the cortex, and ischemia sometimes occurs after glioma resection. However, the clinical significance of postoperative medullary artery–related ischemia has not been well studied. We retrospectively reviewed cases of delayed DWM ischemia after awake craniotomy to elucidate the clinical characteristics, mechanisms, and management of delayed ischemia.

**METHODS:**

We identified 4 cases of intra-axial brain tumors, mainly gliomas, that underwent tumor resection by awake craniotomy at our hospital and developed DWM ischemic symptoms after surgery, despite no worsening of neurological symptoms at the end of surgery.

**RESULTS:**

Four patients (3 men and 1 woman) presented with glioblastoma, oligodendroglioma, astrocytoma, and brain metastasis. The median age at surgery was 47.5 years (41-73 years). The tumors were located in the watershed area in the frontal lobe (*n* = 2) and the parietal lobe (*n* = 2), all of which were left-sided (*n* = 4). DWM ischemic symptoms, such as motor dysfunction, aphasia, dysarthria, and dysgraphia, developed at an average of 24 hours (14-48 hours) after resection by awake craniotomy. All 4 patients showed symptom improvement within a week after surgery and completely recovered within a month.

**CONCLUSION:**

DWM ischemia is caused by sacrifice of the medullary artery, which feeds the tumor and adjacent brain tissue during tumor resection, and should be considered when delayed aphasia or paralysis occurs postoperatively. These symptoms are often transient and recovery usually occurs. Tumors located in the frontal or parietal lobes, particularly in the watershed area, should be carefully monitored for postoperative ischemia.

**ABBREVIATIONS:**
DWM: deep white matter
GTR: gross total resection
IDH: isocitrate dehydrogenase.

Awake surgery is an essential strategy for preserving neurological function during surgery for low-grade gliomas, especially in the dominant hemisphere; however, recent studies have reported that awake surgery is also useful in high-grade glioma, glioblastomas, and metastatic tumor surgeries.^1-3^ In awake surgery, patients can be monitored intraoperatively for the emergence of symptoms, and the surgery can be accomplished without worsening the neurological findings. The difficulty of brain tumor surgery varies depending on its pathology, tumor size, and localization, and the morbidity rate is reported to be 24.2% to 30.7%, in general.^4-6^ In particular, postoperative ischemia should be avoided as much as possible because it can disturb a patient's basic functions, such as limb movement or verbal communication, and lead to a decline in postoperative activities of daily living.^7^

Postoperative deep white matter (DWM) ischemia can sometimes occur after tumor resection. Postoperative ischemia is reported to occur in 12.5% to 61.8% of patients after glioma resection, and 5.4% to 18.2% are reported to be symptomatic.^8-13^ Gliomas generally originate from the white matter and are supplied by the medullary arteries of the cortex. Therefore, tumor resection and damage to this artery cause DWM ischemia.^14^ There are a few reports that DWM ischemia, especially after glioma surgery, is caused by the sacrifice of the long insular or medullary artery.^12,13^ However, the clinical significance of postoperative ischemia caused by the medullary artery damage has not been well studied. In addition, symptomatic ischemia at the end of awake surgery can be explained by intraoperative vascular injury; however, delayed ischemic symptoms occasionally develop despite the absence of symptoms at the end of surgery. In this study, we report 4 cases of delayed DWM ischemia after awake craniotomy. This is the first case series to unravel the clinical characteristics, mechanisms, and management of DWM ischemia after awake craniotomy.

## METHODS

We retrospectively examined our clinical records and found 4 patients who underwent awake craniotomy at our hospital from 2015 to 2022 and developed delayed DWM ischemic symptoms after surgery, despite no worsening of neurological symptoms at the end of surgery compared with that in the preoperative period. Through database abstraction and medical record review, patient age, sex, presenting symptoms, lesion location, and preoperative and postoperative imaging data were collected. All histopathological diagnoses were made by board-certified pathologists at our institution. This study was approved by our institutional review board, which waived the need for informed consent because it deemed this a minimum-risk retrospective study.

## RESULTS

### Patient Characteristics

Three men (75%) and 1 woman (25.0%), with a median age at surgery of 47.5 years (41-73 years; Table), were included in this study. The pathological diagnosis based on the 5th edition of World Health Organization classification of Central Nervous System tumors was glioblastoma isocitrate dehydrogenase (IDH)–wild type, grade 2 oligodendroglioma, grade 3 astrocytoma IDH-mutant, and brain metastasis. Tumors were located in the watershed area in the frontal (50%, *n* = 2) and parietal lobes (50%, *n* = 2), and all of them were left-sided (100.0%, *n* = 4), which is an indication for awake craniotomy. All patients underwent awake craniotomy with an inside-out debulking technique; gross total resection (GTR) was performed in 3 cases (75.0%) and 90% resection in 1 case (25.0%). Neuromonitoring, including motor-evoked potential and somatosensory-evoked potential, was not performed during awake craniotomy in any of the cases. On average, DWM ischemic symptoms, such as motor dysfunction, aphasia, dysarthria, and dysgraphia, developed 24 hours (14-48 hours) after surgery. All the patients were treated with intravenous edaravone or hydration. All 4 patients showed symptom improvement within a week after surgery. The Median Karnofsky Performance Status and the median modified Rankin Scale (mRS) scores before surgery were 85 and 1, respectively. In 3 patients (75.0%), mRS at discharge was the same as that before surgery. One patient (25.0%) developed a slight residual neurological deficit, but the neurological findings were almost completely resolved 1 month after surgery.

**TABLE.** Clinical Characteristics of Our 4 Cases

Characteristics		
Age at surgery, y, median (range)	47.5	(41-73)
Female/male (%)	1/3	(25.0/75.0)
Pathological diagnosis (%)		
Glioblastoma, IDH-wild type	1	(25.0)
Oligodendroglioma, IDH-mutant, grade 2	1	(25.0)
Astrocytoma, IDH-mutant, grade 3	1	(25.0)
Brain metastasis	1	(25.0)
Location (%)		
Frontal	2	(50.0)
Parietal	2	(50.0)
Laterality (%)		
Left	4	(100.0)
Right	0	(0.0)
Tumor resection rate (%)		
GTR	3	(75.0)
PR	1	(25.0)
Postoperative ischemic symptom onset, h, mean (range)	24.0	(14-48)
Karnofsky Performance Status score before surgery, median (range)	85	(80-100)
Modified Rankin Scale score before surgery, median (range)	1	0-2
Modified Rankin Scale change		
No change	3	(75.0)
Decline	1	(25.0)

GTR, gross total resection; IDH, isocitrate dehydrogenase; PR, partial resection.

### Case Illustration

#### Patient 1

**FIGURE 1.**
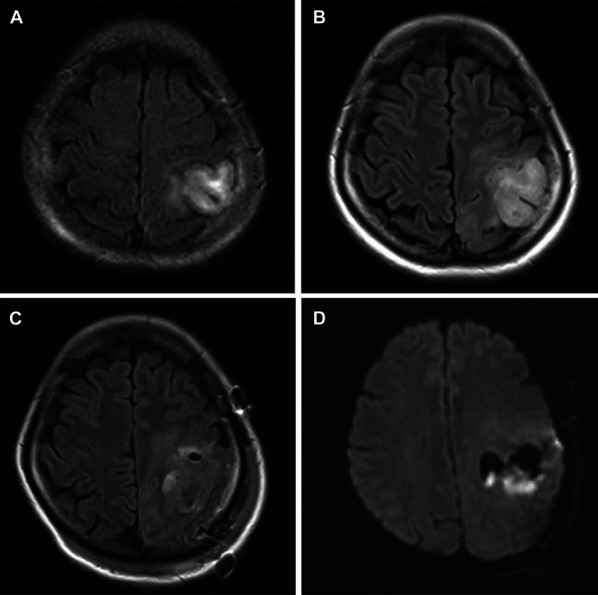
MRI of a 37-year-old woman showing a left parietal brain tumor. **A**, She was asymptomatic and incidentally diagnosed. The patient was followed up in an outpatient clinic for 11 years; **B**, the tumor shows gradual expansion. We performed awake craniotomy. **C**, Image showing almost 90% tumor removal. Nineteen hours after surgery, sensory disturbances in her right hand, dysarthria, and dysgraphia appeared. **D**, Diffusion-weighted MRI showing cerebral ischemia in the deep white matter.

A 37-year-old woman, a medical doctor, was incidentally diagnosed with an asymptomatic left parietal brain tumor that was suspected to be a low-grade glioma (Figure 1A). She refused surgical resection and was followed up at an outpatient clinic for 11 years; however, the tumor showed gradual expansion (Figure 1B), and at the age of 48 years, she developed slight motor dysfunction in her right hand. Because the tumor was located in an eloquent area, we performed an awake craniotomy. Intraoperative monitoring was performed to identify the central sulcus and motor and sensory areas. DWM stimulation was also performed at the time of tumor resection and was stopped when dyscalculia appeared. At the end of awake craniotomy, there was no worsening of neurological deficits compared with the preoperative findings. Almost 90% of the tumor was removed (Figure 1C), and the pathological diagnosis of grade 2 oligodendroglioma, IDH-mutant, and 1p/19q codeletion was obtained. After surgery, the patient was admitted to the intensive care unit (ICU) without any significant changes. However, 19 hours after surgery, sensory disturbances in her right hand, dysarthria, and dysgraphia appeared. Diffusion-weighted magnetic resonance imaging (MRI; DWI-MRI) revealed cerebral ischemia in the DWM (Figure 1D). One week after the surgery, her symptoms gradually improved. She returned to her work as a physician and worked normally 1 month after the surgery.

#### Patient 2

**FIGURE 2.**
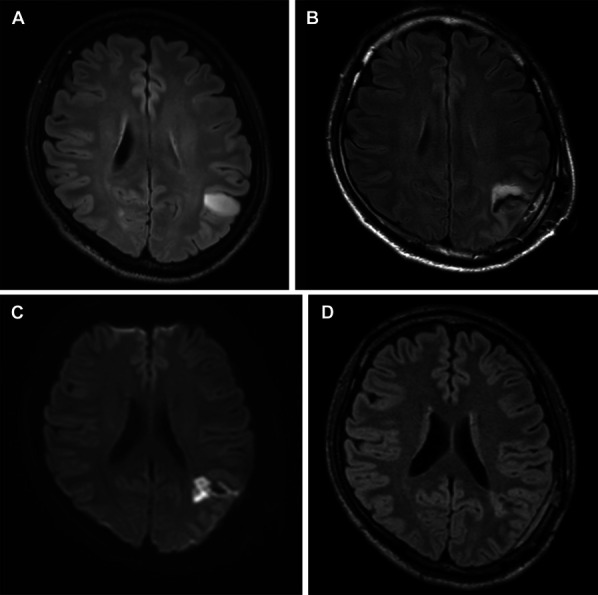
**A**, MRI of a 41-year-old asymptomatic man showing a left parietal brain tumor. **B**, Awake craniotomy to achieve gross total resection is performed (postoperative exudate fluid gives the appearance of high FLAIR signal but not residual tumor). Forty-eight hours after surgery, paraphasia appeared. **C**, DWI-MRI shows cerebral ischemia in the deep white matter; **D**, without a high-intensity area on FLAIR image corresponding to an ischemic lesion. FLAIR, fluid-attenuated inversion recovery.

A 41-year-old man presented with an asymptomatic left parietal brain tumor suspected to be a low-grade glioma on magnetic resonance imaging (MRI; Figure 2A). At the end of tumor resection by awake surgery, there was no worsening of neurological findings compared with the preoperative status. GTR was performed (Figure 2B), and the pathological diagnosis of grade 3 astrocytoma with an IDH-mutant was made. After surgery, the patient was admitted to the ICU without any significant changes and was moved to the general ward the day after the surgery. However, paraphasia appeared 48 hours after surgery, despite the absence of diffuse-weighted imaging (DWI) high signal on MRI the day after surgery. DWI-MRI showed cerebral ischemia in the DWM without a high-intensity area on fluid-attenuated inversion recovery images corresponding to ischemic lesions (Figure 2C and 2D); therefore, we started intravenous edaravone.^15^ Three days after surgery, the patient recovered completely; thus, his resulting symptoms were transient.

#### Patient 3

**FIGURE 3.**
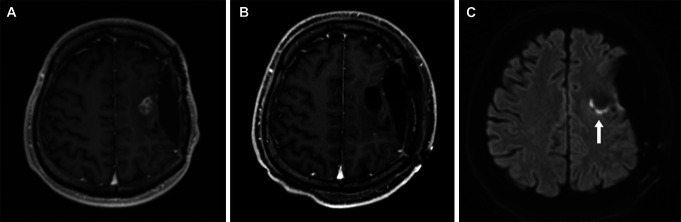
MRI of a 47-year-old man after treated with radiation, chemotherapy, and **A**, tumor treating fields showing progressive disease of glioblastoma. **B**, Awake craniotomy to achieve gross total resection is performed. Fourteen hours after surgery, the worsening of right-sided paralysis appeared. **C**, DWI-MRI showing cerebral ischemia in the deep white matter (arrow).

A 47-year-old man experienced a seizure a year ago, and his previous physician indicated a brain tumor. The patient underwent craniotomy for tumor resection and was diagnosed with an IDH–wild type glioblastoma. He was treated with radiation, chemotherapy, and tumor treating fields; however, recurrence occurred 9 months postoperatively. He was referred to our hospital for further treatment; however, the tumor progressed (Figure 3A); therefore, we performed tumor resection by awake craniotomy. Before surgery, the patient had mild right-sided paralysis; however, at the end of the surgery, there was no worsening of the neurological findings compared with the preoperative findings. GTR was performed (Figure 3B), and the pathological diagnosis of glioblastoma recurrence and IDH-wild type was made. After the surgery, the patient was admitted to the ICU without any significant changes. However, 14 hours after surgery, right hemiparesis worsened. DWI-MRI showed cerebral ischemia in the DWM (Figure 3C), and the patient was started on intravenous edaravone. One week after surgery, his symptoms recovered to the preoperative level.

#### Patient 4

**FIGURE 4.**
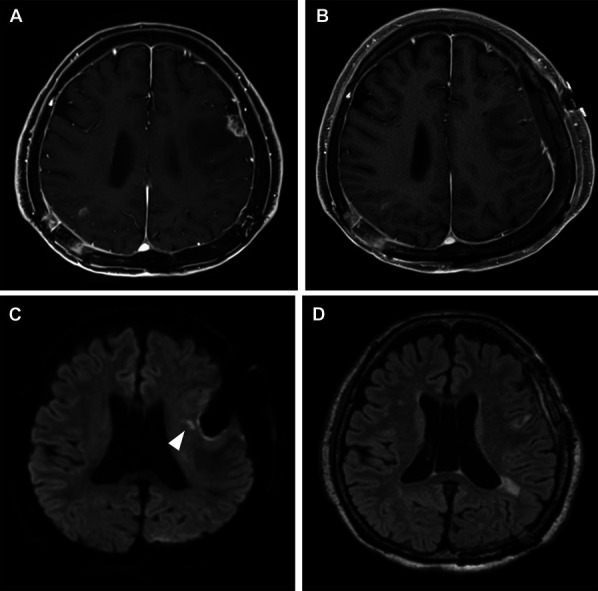
**A**, MRI of a 73-year-old man after craniotomy and stereotactic radio surgery showing recurrent brain metastasis. **B**, Awake craniotomy to achieve gross total resection is performed. Fifteen hours after surgery, motor aphasia appeared. **C**, DWI-MRI showing cerebral ischemia in the deep white matter (arrowhead) without a high-intensity area on **D**, the fluid-attenuated inversion recovery image corresponding to the ischemic lesion.

A 73-year-old man was diagnosed with lung cancer 12 years prior and underwent craniotomy for brain metastasis originating from lung cancer in the left occipital and right parietal lobes. He underwent stereotactic radiosurgery for the left frontal lesion 4 years prior, and recurrence was suspected (Figure 4A, and the patient was urgently admitted to our hospital because of the appearance of motor aphasia. We performed tumor resection by awake craniotomy to prevent worsening of the neurological findings. At the end of the surgery, there was no worsening of the neurological findings compared with the preoperative findings. GTR was performed (Figure 4B), and the pathological diagnosis of brain metastasis originating from lung cancer was obtained. After the surgery, the patient was admitted to the ICU without any significant changes. However, 15 hours after surgery, motor aphasia appeared. DWI showed cerebral ischemia in the DWM without a high-intensity area on fluid-attenuated inversion recovery images corresponding to ischemic lesions (Figure 4C and 4D), and intravenous edaravone was initiated. Two days after surgery, the patient's symptoms recovered completely; thus, the symptoms were transient.

## DISCUSSION

### Ischemia After Tumor Resection

The aim of surgical treatment of gliomas was maximal surgical resection without neurological deficits.^16^ To achieve this, intraoperative neuropsychological monitoring, such as awake surgery, is essential to preserve neurological function.^8^ Postoperative ischemia is one of the most critical postoperative complications, which are reported to occur in 12.5% to 61.8% of patients after glioma resection, and 5.4% to 18.2% are reported to be symptomatic.^8-13^ These reports include surgeries for high-grade and low-grade gliomas, so it is difficult to discuss them in general; however, it is believed that brain ischemia during surgery results from direct vascular damage and coagulation, vasospasm, and kinking of arteries by retraction of the brain,^8,17^ leading to acute ischemia after surgery. Such cerebral infarctions occur most frequently in the insula, temporal lesions, and recurrent tumor.^8-10,16^ This is because the insula is notorious for its high risk of morbidity owing to its complex vascular supply, which is mainly based on frequent perforating arteries with no collateral flow.^8,18^ However, radiogenic and postoperative tissue changes can contribute to a higher risk of morbidity in patients with recurrent tumors.^16^

Despite these reports, there have been no reports of delayed ischemia after glioma surgery. In a review of delayed ischemia after meningioma resection, the mean time of onset was 9.7 days, and cerebral vasospasm was thought to be the mechanism behind delayed ischemia.^19^ However, intramedullary tumors, such as glioma and brain metastasis, where the tumor extends into the cerebral white matter, are more likely to damage normal brain function than extramedullary tumors such as meningiomas, and the mechanism of postoperative delayed ischemia could be considered to be different accordingly.

### Medullary Artery and DWM Ischemia

**FIGURE 5.**
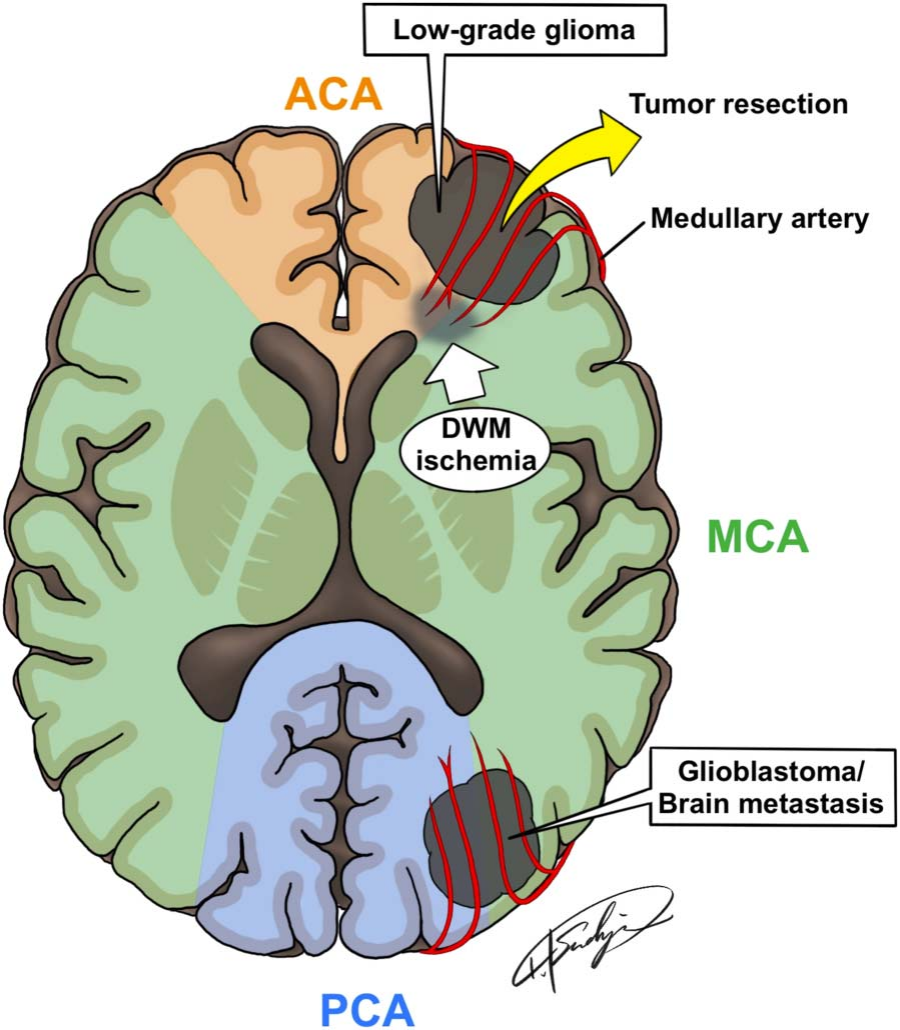
The medullary artery supplies the blood for glioma and deep white matter, and tumor resection with coagulation of the medullary artery results in ischemic complications, such as paralysis or aphasia because of damage of the nerve tract running in the deep white matter. ACA, anterior cerebral artery; MCA, middle cerebral artery; PCA, posterior cerebral artery.

In insular glioma surgery, the long insular and medullary arteries are the distributing arteries to the corona radiata, which is important for postoperative movement disorders.^12,13^ In insular glioma surgery and tumors extending into the cerebral white matter, the medullary artery is important as the feeding vessel of the DWM. The medullary arteries are long-ended arteries that arise at a right angle from the pial arteries, penetrate the cerebral cortex, and enter the white matter.^14^ The medullary artery also supplies blood to the glioma (Figure 5), and tumor resection through the medullary artery results in ischemic complications, which can cause paralysis or aphasia because of damage to the nerve tract running in the DWM.

In our 4 cases, no obvious intraoperative major vessel damage was observed; however, DWM ischemia developed postoperatively. We cannot prove or confirm that the medullary artery was difficult to visualize on imaging studies^14^; however, it is believed that the medullary artery was sacrificed through coagulation of small arteries during tumor resection. Thus, the medullary artery is an important vessel in intramedullary tumor resection because it is thin and can be easily damaged and can sometimes lead to symptoms of DWM ischemia.

The cause of the delayed onset of symptoms or transient improvement cannot be definitively determined. The transient course of the condition may be attributed to a number of potential causes, including venous congestion, arterial injury of adjacent pia, and cerebral vasospasm. Another possible cause is that the medullary artery is a terminal artery,^14,18^ but there may be some collateral flow, and ischemia may be caused by decreased perfusion, such as postoperative dehydration or temporary arterial hypotension from other causes. In our 4 cases, all tumors were located between the anterior cerebral artery and middle cerebral artery in the frontal lobe and the middle cerebral artery and posterior cerebral artery in the parietal lobes; thus, the ischemia can also be explained as hemodynamic ischemia because these areas are most likely to be in the watershed region.^20,21^ Because tumor location is reported to be a predictive factor for postoperative stroke,^8^ we suspect that tumors located in the watershed area of the frontal or parietal lobes are at risk of DWM ischemia and should be carefully monitored postoperatively. Furthermore, DWM ischemia may be particularly prone to manifestation in gliomas of the dominant hemisphere, which are the target of awake craniotomy.

### Management and Prognosis

Postoperative dehydration and anemia should be corrected to prevent DWM ischemia. We administered intravenous edaravone to 3 of our 4 patients and intravenous infusions to prevent dehydration. Edaravone has been shown to be effective in treating acute cerebral infarction, especially within 24 hours of onset.^15,22^ All patients showed rapid improvement in neurological symptoms, and 3 of the 4 patients had the same mRS scores at discharge and preoperatively. The effects of edaravone and infusion in our cases are not certain; however, our cases suggest that the symptoms of delayed DWM ischemia may be transient and recoverable. However, the prognosis of this pathology should be studied in the future with the accumulation of more cases.

### Limitations

There are some limitations to our study. Transient DWM ischemia is thought to be caused by the sacrifice of the medullary artery; however, it has not been objectively proven by high-resolution computed tomography angiography or angiogram. Moreover, the power of the study is limited by the lack of reported cases of delayed DWM ischemia after awake surgery, which prevented a comparison with previous literature. We hope that further accumulation of such cases will enable a more detailed analysis of its clinical evolution and the most appropriate management in the future.

## CONCLUSION

This study is the first to illustrate the clinical significance of delayed DWM ischemia after tumor resection by awake craniotomy, which is a rare but important pathology. If delayed aphasia or paralysis occurs after tumor resection, DWM ischemia because of intraoperative injury to the medullary artery should be considered and treatment for ischemia should be initiated soon. In our case, these symptoms were often transient and resolved. The lesson, therefore, is that tumors located in the frontal or parietal lobes, particularly in the watershed area, should be carefully monitored for postoperative ischemia.

## COMMENTS

Postoperative deep white matter ischemia after glioma resection can be a challenging clinical complication that can be due to direct vascular injury, vasospasm, venous congestion, or prolonged brain retraction.

The authors report a single-site retrospective study on 4 cases of intra-axial brain tumors that underwent tumor resection by awake craniotomy by an inside-out debulking technique where each case developed postoperative symptoms and MRI findings consistent with deep white matter (DWM) ischemia. All of these cases presented in a delayed fashion (after 24 hours) despite no neurological symptoms at the immediate conclusion of surgery. The cohort of tumors were located in dominant watershed areas, and all improved in a few weeks after surgery.

Despite no clear evidence of intraoperative major vessel injury, the authors propose that the medullary artery injury may contribute to DWM pathophysiology in dominant watershed white matter tracts. Although there is no direct evidence of medullary artery injury, the proposed mechanism of DWM is certainly interesting and worth reporting. Delayed onset of symptoms in these cases could also be related to venous congestion, cerebral vasospasm, hypovolemia, and cerebral edema from surgical manipulation. Further validation of the pathophysiology would be helpful using high-resolution computed tomography angiography (CTA) or angiography in relevant cases. Additionally, surgical strategies to avoid and mitigate DWM could be considered including the use of intraoperative neuromonitoring, reducing vasospasm with intracavitary papaverine and permissive hypertension. Future prospective larger case series assessing the utility of postoperative adjuncts such as edaravone in the setting of DWM may be useful.

**Christian K. Ramsoomair, Manav Daftari, and Ashish H. Shah**

*Miami, Florida, USA*
